# Dual Targeting of Galanin Receptor 3 Signaling and Redox Homeostasis Enhances Photoreceptor Survival in Retinas of *rd10* Mice

**DOI:** 10.3390/antiox15091073

**Published:** 2026-08-27

**Authors:** Maria Azam, Mingda Liu, Beata Jastrzebska

**Affiliations:** 1 Department of Pharmacology, School of Medicine, Case Western Reserve University, 10900 Euclid Ave., Cleveland, OH 44106, USA; azamm3@ccf.org (M.A.); mxl1118@case.edu (M.L.); 2 Cleveland Center for Membrane and Structural Biology, Case Western Reserve University, 10900 Euclid Ave., Cleveland, OH 44106, USA

**Keywords:** galanin receptors, neuroinflammation, oxidative stress, photoreceptor, quercetin, retinal degeneration, retinitis pigmentosa

## Abstract

Retinitis pigmentosa (RP) is a genetically heterogeneous group of inherited retinal degenerative disorders characterized by progressive photoreceptor loss and vision impairment, for which broadly applicable mutation-independent therapies remain limited. To examine the therapeutic potential of combined galanin receptor 3 (GALR3) inhibition and antioxidant therapy in a mutation-independent context, we utilized the *rd10* mouse model of RP. We first evaluated the effects of individual treatments with the GALR3 antagonist SNAP-37889 and the antioxidant quercetin, followed by a combined treatment regimen to determine whether simultaneous targeting of neuroinflammatory and oxidative stress pathways provides enhanced retinal protection. Treatment efficacy was assessed using functional and morphological analyses, including electroretinography (ERG) to measure retinal function, optical coherence tomography (OCT) to evaluate retinal structure in vivo, and histological and immunohistochemical analyses to quantify photoreceptor survival and markers of retinal oxidative stress and inflammation. Although the expression levels of individual inflammatory and oxidative stress markers did not consistently exhibit additive responses, the combined treatment produced greater photoreceptor survival and preservation of photopic retinal function than either monotherapy alone. These findings support the hypothesis that simultaneous modulation of oxidative stress and neuroinflammation provides greater neuroprotective benefits, establish a foundation for the development of mutation-independent therapeutic strategies for RP, and identify GALR3 as a promising therapeutic target.

## 1. Introduction

Retinitis pigmentosa (RP) represents a clinically and genetically heterogeneous group of inherited retinal degenerative disorders that ultimately lead to blindness [1,2]. To date, mutations in more than 70 genes have been identified as causative factors, emphasizing the complexity of its genetic landscape [3]. Despite decades of progress in understanding the genetic basis of RP, there remains no effective treatment capable of halting or reversing disease progression. While genetic mutations initiate the degenerative process, growing evidence indicates that secondary cellular stress responses play a critical role in disease progression and photoreceptor demise. Dysregulated Ca^2+^ homeostasis, oxidative stress, neuroinflammation, and mitochondrial dysfunction act as key mediators that amplify the effects of the primary mutation, ultimately converging on photoreceptor apoptosis and retinal degeneration [4,5]. These secondary pathways are now recognized as major obstacles to developing broadly effective therapies and represent promising targets for mutation-independent treatment strategies.

A growing body of preclinical research demonstrates that pharmacological interventions with antioxidant and anti-inflammatory properties can attenuate photoreceptor loss and preserve visual function in several models of RP, including *rd1*, *rd10*, Q344ter, and P23H rhodopsin [6,7,8,9,10]. We have shown that among these agents, quercetin, a naturally occurring flavonoid, can reduce oxidative damage, suppress microglial activation, and rescue photoreceptors in both the P23H rhodopsin mutant mouse model and mice vulnerable to bright light insult [8,11]. Treatment with quercetin improved retinal structure and function, supporting its dual antioxidant and anti-inflammatory potential in degenerative retinal conditions.

In addition, we recently identified galanin receptor 3 (GALR3), a G_i_-coupled G protein-coupled receptor (GPCR) that has been implicated in stress-associated signaling and neuroinflammatory responses within the central nervous system [12,13] as a key regulator of microglial activation and retinal inflammation in degenerating retinas of P23H rhodopsin mice and in mice with retinal degeneration induced by bright light [14,15]. Pharmacological and genetic inhibition of GALR3 signaling delayed photoreceptor death and preserved retinal integrity in these models, highlighting its therapeutic relevance and suggesting that GALR3 signaling contributes to secondary inflammatory responses that may promote photoreceptor degeneration irrespective of the initiating genetic or environmental insult.

Building on these findings, we hypothesized that targeting GALR3 signaling may represent a mutation-independent strategy for the treatment of RP. To test this hypothesis in a genetically distinct form of RP, we used *rd10* mice, a well-characterized model of autosomal recessive RP (arRP) caused by a mutation in the phosphodiesterase 6b (*Pde6b*) gene. Unlike the P23H rhodopsin model, in which degeneration is initiated by a mutant rhodopsin, *rd10* photoreceptor degeneration results from impaired PDE6B function and dysregulated cGMP signaling, providing an important opportunity to determine whether GALR3-dependent neuroinflammation represents a convergent pathway contributing to photoreceptor degeneration across genetically diverse forms of RP. Thus, the use of the *rd10* model was chosen to determine whether GALR3-dependent neuroinflammation can be therapeutically targeted despite fundamentally different disease-initiating mechanisms. Because oxidative stress is also a prominent feature of *rd10* retinal degeneration and previous studies have shown that flavonoids, including quercetin, can reduce oxidative stress in these mice [9,16], we further hypothesized that simultaneous targeting of neuroinflammation through GALR3 inhibition and oxidative stress through quercetin could provide greater neuroprotection than either intervention alone. Accordingly, we investigated whether inhibition of GALR3 protects against photoreceptor degeneration in *rd10* mice and whether combined treatment with a GALR3 antagonist and quercetin produces an enhanced effect on retinal structure and function. This approach allowed us to test whether dual modulation of distinct secondary stress pathways can enhance neuroprotection and support GALR3 inhibition as a potential mutation-independent therapeutic strategy for RP.

## 2. Materials and Methods

### 2.1. Chemicals and Reagents

A BCA Protein Assay Kit was obtained from Thermo Fisher Scientific (Pittsburg, PA, USA). Dimethylsulfoxide (DMSO) and EDTA-free protease inhibitor tablets were purchased from Sigma (St. Louis, MO, USA). Fluoromount-G slide mounting medium was purchased from SouthernBiotech (Birmingham, AL, USA). Polyvinylidene difluoride (PVDF) membrane was obtained from Millipore (Burlington, MA, USA). RIPA lysis buffer was purchased from Thermo Fisher Scientific. The GALR3 antagonist SNAP-37889 was purchased from Alomone Labs, Ltd. (Jerusalem, Israel) (PubChem CID 1471834). Peanut agglutinin (PNA) and Alex Fluor 488-conjugated streptavidin were obtained from Vector Laboratories (Newark, CA, USA). Luna Universal qPCR Master Mix was purchased from New England Biolabs (Ipswich, MA, USA).

### 2.2. Animals

In this study, we used homozygous retina degeneration 10 (B6.CXB1-*Pde6b^rd10^/J*) mice (Research Resource Identifier, RRID:IMSR_JAX:004297) (Jackson Laboratory, Bar Harbor, ME, USA), which possess a spontaneous missense point mutation in the cGMP phosphodiesterase 6B, rod receptor, beta polypeptide (*Pde6b*), also known as *rd10* mice, as a model of RP and wild-type (WT) C57BL/6J mice (RRID:IMSR_JAX:000664) (Jackson Laboratory, Bar Harbor, ME, USA). Both male and female mice were used in all experiments. Mice were housed in the Animal Resource Center at the School of Medicine, Case Western Reserve University (CWRU), and WT C57BL/6J mice were maintained in a 12 h light/dark cycle all the time, while *rd10* mice were kept in the dark until postnatal day 21 (P21), then they were moved to a room with a 12 h light/dark cycle. All the procedures involving mice and experimental protocols received approval from the Institutional Animal Care and Use Committee (IACUC) at CWRU and comply with the Animal Welfare Act guidelines and the ARRIVE guidelines. Animals were treated according to guidelines and recommendations of both the American Veterinary Medical Association Panel on Euthanasia and the Association for Research in Vision and Ophthalmology, as well as the National Eye Institute Animal Care and Use Committee (NEI-ASP 682) in an effort to minimize their suffering.

### 2.3. Pharmacological Treatment

Beginning at postnatal day 21 (P21), *rd10* mice were treated every other morning for two weeks either with GALR3-specific antagonist SNAP-37889 [17] (10 mg/kg body weight (b.w.)), quercetin (10 mg/kg), their combination, or vehicle (50% DMSO/PBS) as previously established [15]. Treatments (six in total) were administered intraperitoneally (i.p.). Mice were analyzed at P33 following established protocols [8,14,18,19]. Retinal structure was examined with SD-OCT (*n* = 6 mice per group), and retinal function was evaluated by ERG (*n* = 5–11 mice per group). Prior to each procedure, mice were anesthetized with a cocktail containing ketamine (20 mg/mL) and xylazine (1.75 mg/mL) at a dose of 4 µL/g b.w. For histological and immunohistochemical retinal analyses, eyes were collected from euthanized mice (*n* = 6 per group).

### 2.4. In Vivo Retina Imaging

Retinal degeneration associated with the *Pde6b* mutation was evaluated in *rd10* mice following pharmacological inhibition of GALR3 with SNAP-37889, treatment with antioxidant quercetin, their combination, or vehicle at P33 using ultrahigh-resolution spectral-domain optical coherence tomography (SD-OCT; Bioptigen, Morrisville, NC, USA). Imaging was performed with an A-scan/B-scan ratio of 1200 lines. B-mode scans were acquired at 0° and 90° orientations, and five consecutive frames were averaged to improve image quality. Quantitative analysis of retinal structure was based on the thickness of the outer nuclear layer (ONL), measured at 0.5 mm from the optic nerve head (ONH). WT C57BL/6J mice were used as a control. Six mice were used per experimental group.

### 2.5. Histological Analysis

Eyes collected from euthanized mice were immersion-fixed in 0.5% glutaraldehyde and 2% paraformaldehyde (PFA) in phosphate-buffered saline (PBS) for 24 h at room temperature (RT) on a rotator. Subsequently, the eyes were transferred to 1% PFA for post-fixation over 48 h at RT. Paraffin-embedded eyes were sectioned at 5 µm and stained with hematoxylin and eosin (H&E). Retinal morphology was visualized and analyzed using a ZEISS Axio Scan.Z1 slide scanner (Carl Zeiss Microscopy GmbH, Jena, Germany) with ZEN 3.2 software (Blue edition).

### 2.6. Immunohistochemistry

Mouse eyes fixed in 2% paraformaldehyde (PFA) for 72 h at room temperature (RT) were used to prepare 8 μm thick cryosections. Prior to immunolabeling, sections were blocked for 1 h at RT in PBS containing 10% normal goat serum (NGS) and 0.3% Triton X-100. Sections were then incubated with the primary antibody overnight at 4 °C. The following day, slides were washed three times with PBS (5 min each) and subsequently incubated with the secondary antibody for 2 h at RT. Rod photoreceptors were identified using a 1D4 anti-rhodopsin antibody, while cone photoreceptors were visualized with biotinylated peanut agglutinin (PNA) followed by Alexa Fluor 488-conjugated streptavidin. Alexa Fluor 555-conjugated goat anti-mouse or anti-rabbit IgG (1:400) was used as the secondary antibody for all immunolabeling. GFAP-positive Müller glia were labeled using a rabbit polyclonal anti-GFAP antibody (1:200), and IBA-1-positive microglia were detected with a rabbit polyclonal anti-IBA-1 antibody (1:100) (see Table 1). Cell nuclei were counterstained with DAPI, and slides were mounted with Fluoromount-G (SouthernBiotech).

### 2.7. Electroretinography

Scotopic and photopic ERG responses were recorded from both eyes of each mouse using a Celeris rodent ERG system coupled with Espion software, Version 6 (Dyagnosys, LLC, Lowell, MA, USA). ERG measurements were performed at P33 in *rd10* mice treated with either SNAP-37889, quercetin, their combination, or vehicle and in WT C57BL/6J control mice. Each experimental group included ten to eleven mice and WT control five mice. Amplitude values for both a- and b-waves were quantified and expressed as mean ± standard error (SEM) for each experimental group.

### 2.8. Retinal Flat Mount

Mouse eyes were enucleated and fixed in 4% paraformaldehyde in PBS at 4 °C. A small incision was made in the sclera to facilitate fixation. After removal of extraocular muscles and connective tissue under a dissection microscope, the anterior segment (cornea, iris, and lens) was excised. The eyecup was then divided into four quadrants extending from the periphery to the optic nerve head to prepare retinal flat mounts. For RPE-choroid flat mounts, the retina was carefully detached. Flat mounts were washed thoroughly in PBS and incubated for 24 h at 4 °C, followed by blocking for 24 h at 4 °C in PBST composed of PBS, 0.5% Triton X-100, and 0.05% Tween-20, and containing 5% NGS and 5% bovine serum albumin (BSA). Tight junctions were detected using a polyclonal anti-ZO-1 antibody (1:200), and albumin was labeled using a FITC-conjugated polyclonal anti-albumin antibody (1:100), both incubated overnight at 4 °C (see Table 1). After washing four times in PBS (30 min each), tissues were incubated with Alexa Fluor 488-conjugated anti-rabbit IgG (1:400) for 3 h at RT or overnight at 4 °C. All antibodies were diluted in PBST containing 1% NGS. Following labeling with secondary antibody, tissues were washed, counterstained with DAPI for 30 min, washed again, and mounted in Fluoromount-G (SouthernBiotech) for imaging under a fluorescence microscope.

### 2.9. Quantification of Gene Expression

The selected gene expression analyses were performed on mouse retinas (*n* = 6 mice per treatment group). Total RNA was extracted using the RNeasy Mini Kit (Qiagen, Venlo, The Netherlands) according to the manufacturer’s instructions and treated with DNase I to remove genomic DNA contamination. RNA concentration and purity were assessed using a NanoDrop spectrophotometer (Thermo Fisher Scientific). Complementary DNA (cDNA) was synthesized from total RNA using the QuantiTect Reverse Transcription Kit (Qiagen) following the manufacturer’s protocol. Real-time quantitative PCR (RT-qPCR) was performed using LUNA SYBR Green Master Mix (NEB) on a StepOnePlus Real-Time PCR System (Applied Biosystems, Waltham, MA, USA). The thermal cycling conditions were as follows: initial denaturation at 95 °C for 3 min, followed by 40 cycles of 95 °C for 20 s and 60 °C for 60 s, with fluorescence acquisition at 60 °C. A melt curve analysis was included to confirm amplicon specificity. *Gapdh* was used as the internal housekeeping gene for normalization. C_t_ values were obtained from the amplification curves using StepOne software version 2.3. Relative gene expression levels were calculated using the comparative 2^−(ΔΔCt)^ method. All primers used in this study are listed in Table 2.

### 2.10. Immunoblotting

Proteins were extracted from retinas. Tissues were mechanically homogenized in RIPA lysis buffer (Thermo Fisher Scientific) supplemented with a protease inhibitor cocktail (Sigma) and incubated for 30 min at 4 °C. The lysates were then centrifuged at 12,000× *g* for 15 min at 4 °C to remove debris. Protein concentrations were determined using the BCA Protein Assay Kit (Thermo Fisher Scientific) with bovine serum albumin as the standard. Equal amounts of protein (50 µg per lane) were separated by SDS-PAGE using 10% or 12% polyacrylamide gels and subsequently transferred onto polyvinylidene difluoride (PVDF) membranes (Millipore). Membranes were blocked with 5% non-fat dry milk in TBST (Tris-buffered saline with 0.1% Tween-20) for 1 h at RT, then incubated overnight at 4 °C with the appropriate primary antibodies. The following day, membranes were washed and incubated with horseradish peroxidase (HRP)-conjugated anti-mouse or anti-rabbit secondary antibodies. Immunoreactive bands were visualized using the ProSignal reagent kit (Genesee Scientific, El Cajon, CA, USA) and the Odyssey Imaging System (LI-COR, Biosciences, Lincoln, NE, USA) according to the manufacturer’s instructions. Actin was used as the loading control.

### 2.11. Statistical Analyses

Immunoblot experiments were repeated at least three times, and band intensities were quantified using ImageJ software 1.54g, National Institute of Health (NIH), Bethesda, MD, USA. Each experiment included both positive and negative controls. Results are expressed as mean ± standard deviation (S.D.), while ERG results are presented as mean ± SEM. Statistical analyses were conducted using GraphPad Prism 10. For multiple group comparisons, one-way or two-way ANOVA with Dunnett’s or Tukey’s post hoc test was employed, whereas two group comparisons were performed using Student’s *t*-test. A significance threshold of *p* < 0.05 was applied. Data acquisition and statistical evaluation were performed independently by separate investigators to maintain objectivity. Different personnel carried out data collection and their statistical analysis.

## 3. Results

### 3.1. Inhibition of GALR3 and Quercetin Treatment Improve Retinal Morphology in rd10 Mice

**Figure 1:**
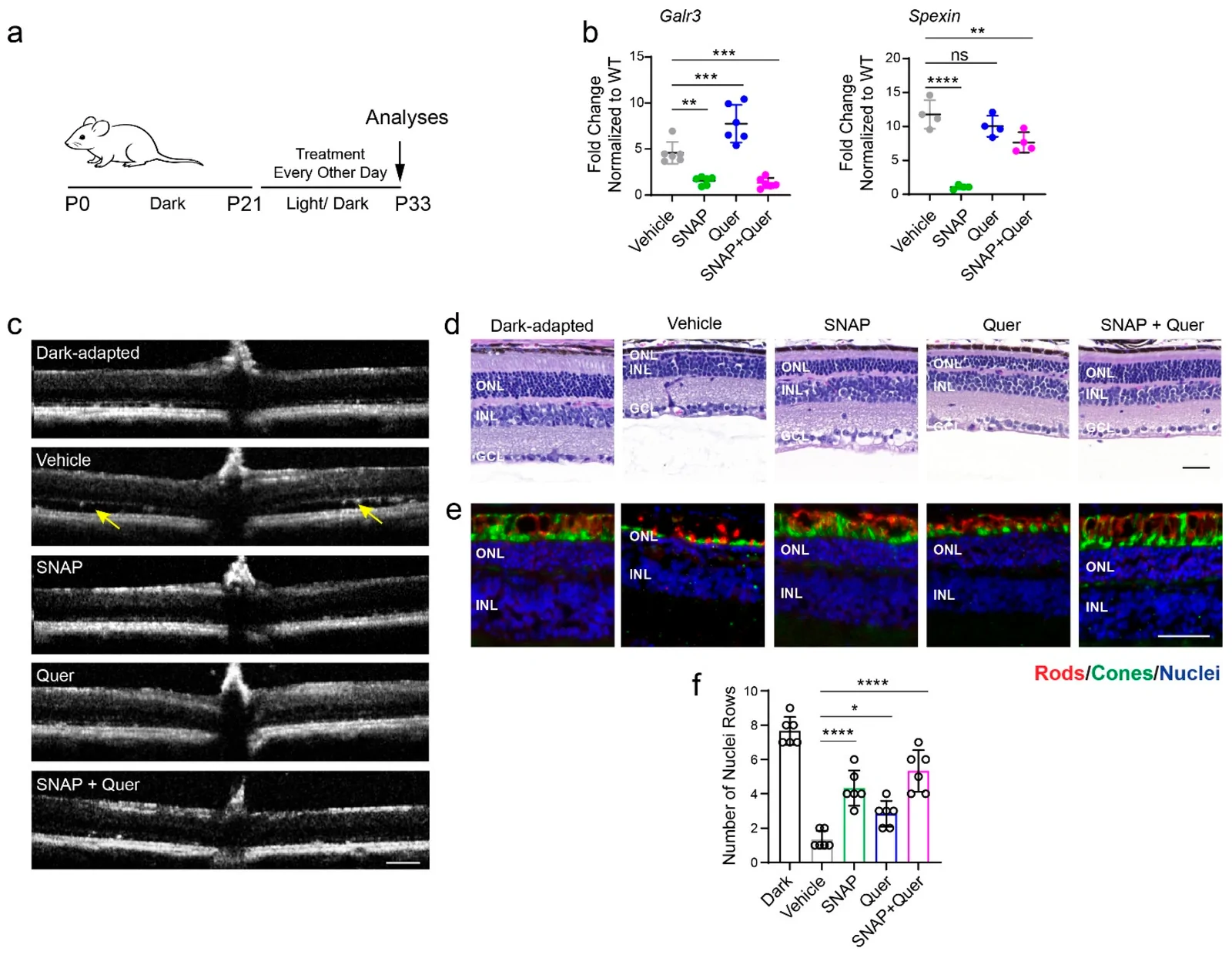
Effects of GALR3 antagonist, quercetin and their combination on the retinal morphology. (**a**) Schematic of experimental design, mouse treatment regimen and analysis. Mice were treated with GALR3 antagonist SNAP-37889, quercetin, or their combination starting at postnatal day 21 (P21) until P33 every other day followed by retinal analyses. (**b**) mRNA expression levels of *Galr3* and *spexin* in retinas of *rd10* normalized to their expression in WT mice detected with RT-qPCR. (**c**) Effects of treatments on retinal morphology examined by SD-OCT. Scale bar 100 µm. Arrows show the retina detachment. (**d**) Effects of treatments on retinal morphology examined by histological analysis. Scale bar 50 µm. (**e**) Immunohistochemistry analysis of rods and cone photoreceptors in retinal cryosections. Rods were labeled with a 1D4 anti-rhodopsin antibody (red) and cones with peanut agglutinin (PNA, green). ONL, outer nuclear layer; INL, inner nuclear layer; GCL, ganglion cell layer. (**f**) Quantification of the ONL thickness at 0.5 mm from the optic nerve head (ONH). Error bars represent standard deviation (S.D.). Statistically significant changes that were calculated with one-way ANOVA and Dunnett’s post hoc test are shown with asterisks. *, *p* < 0.05; **, *p* < 0.01; ***, *p* < 0.001; ****, *p* < 0.0001; ns, not statistically significant changes.

Based on our previous findings implicating GALR3 signaling in retinal degeneration [14,15], we investigated whether GALR3 also contributes to photoreceptor loss in *rd10* mice, a well-established model of RP caused by a mutation in *Pde6b*. To evaluate the therapeutic potential of GALR3 inhibition and antioxidant treatment, *rd10* mice were treated with the GALR3-specific antagonist SNAP-37889, quercetin, or a combination of both agents every other day between postnatal day 21 (P21) and P33, followed by analyses of retinal structure, function and the expression of specific markers (Figure 1a).

First, using RT-qPCR analysis, we confirmed that *Galr3* mRNA expression and the levels of its endogenous agonist, *spexin*, were significantly increased in the retinas of *rd10* mice at P33 compared to age-matched wild-type (WT) controls, consistent with our observations in P23H rhodopsin [15] and light-damage mouse models [14] (Figure 1b). As expected, levels of these markers were reduced in retinas of mice treated with either SNAP-37889 or a combination of SNAP-37889 and quercetin, while quercetin monotherapy had no significant effect on *Galr3* and *spexin* mRNA levels.

Examination of retinal structure using in vivo optical coherence tomography (OCT) imaging revealed that vehicle-treated *rd10* mice maintained under standard 12 h light/12 h dark conditions between P21 and P33 exhibited severe retinal degeneration and extensive retinal detachment at P33 compared to dark-adapted controls (Figure 1c). Remarkably, treatment with SNAP-37889, quercetin, or their combination markedly reduced the extent of retinal detachment. Histological analysis further confirmed pronounced thinning of the outer nuclear layer (ONL) in vehicle-treated mice, with only 1–2 rows of photoreceptor nuclei remaining, whereas dark-adapted controls retained up to 7–9 rows (~60% of the ONL thickness of WT mice [15]). In contrast, mice treated with SNAP-37889, quercetin, or both exhibited 3–7 nuclei rows, demonstrating their protective effects on retinal structure and integrity (Figure 1c–f).

**Figure 2:**
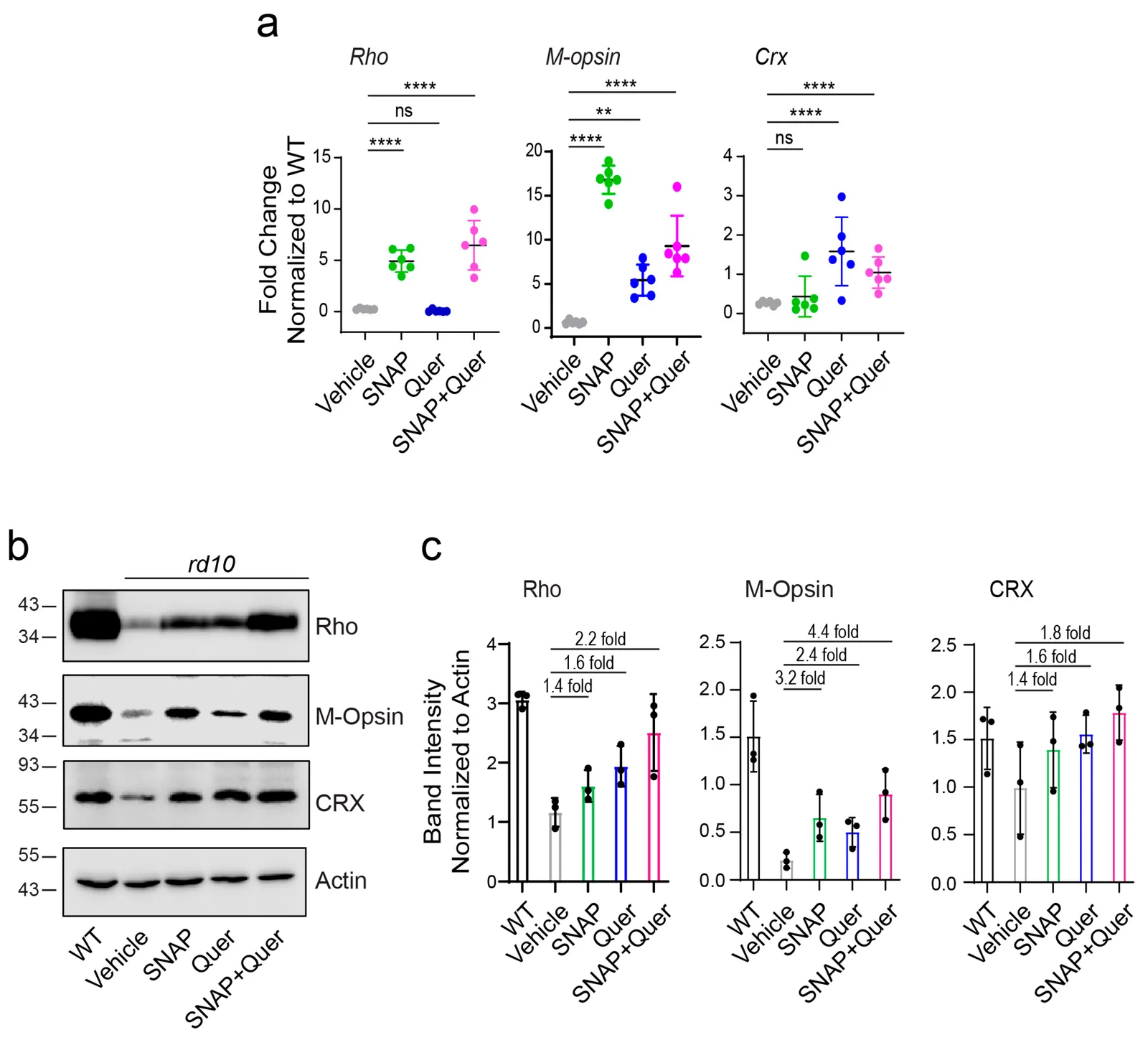
Effects of GALR3 antagonist, quercetin and their combination on the expression of visual receptors and their expression regulator in retinas of *rd10* mice. (**a**) mRNA expression levels of *Rho*, *M-opsin*, and *Crx* detected with RT-qPCR normalized to *Gapdh* and these genes’ expression in WT mice. (**b**) Protein expression levels of Rho, M-opsin, and CRX assessed by immunoblotting. The representative immunoblots are shown. Full immunoblots are shown in Figure S1 in Supplementary Information. (**c**) Quantification of Rho, M-opsin, and CRX band intensities detected in three immunoblots from independent experiments normalized to actin. Fold change was calculated. Error bars represent S.D. Statistical analysis was performed with one-way ANOVA and post hoc Dunnett’s tests. Statistically significant changes are shown with asterisks. **, *p* < 0.01; ****, *p* < 0.0001; ns, not statistically significant changes.

To assess the effects of GALR3 signaling inhibition, quercetin treatment, and their combination on photoreceptor integrity, retinal cryosections were immunolabeled with the 1D4 anti-rhodopsin antibody to visualize rods and biotinylated peanut agglutinin (PNA), which selectively labels cone photoreceptor outer segments (Figure 1e). Immunohistochemical analysis revealed preservation of both rod and cone photoreceptors in treated retinas compared with vehicle-treated controls, with the greatest protection observed following the combination treatment. Consistent with these findings, rhodopsin and M-opsin mRNA and protein levels, assessed by RT-qPCR and immunoblotting, respectively, were markedly reduced in vehicle-treated *rd10* mice compared with WT controls but were significantly increased following treatment. The greatest increases in both rhodopsin and M-opsin expression were observed in the combination treatment groups (Figure 2a–c and Figure S1).

In addition, we examined the expression of cone-rod homeobox (CRX), a master regulator of photoreceptor differentiation and maintenance [20]. CRX levels were markedly reduced in *rd10* mice compared to WT controls. However, treatment with the GALR3 antagonist, quercetin, or their combination significantly upregulated CRX expression, restoring it to levels comparable to those observed in WT mice (Figure 2a–c and Figure S1). Notably, the combination treatment tended to produce higher CRX levels than either monotherapy, although the differences were modest. Together, these findings suggest that GALR3 inhibition and quercetin treatment promote photoreceptor survival while preserving the transcriptional programs required for photoreceptor maintenance in the degenerating retinas of *rd10* mice.

In *rd10* mice, rod photoreceptor degeneration begins as early as P10, and by P30, most rods are lost. Cone degeneration occurs later, around P20, and progresses between P30 and P40. Since our treatments started at P21 when cone photoreceptor degeneration begins, and effects were evaluated at P33, a stage when cone loss is already substantial, we examined whether the interventions could preserve cone photoreceptor survival by quantifying PNA-stained cones in retinal flat mounts. All treatment groups exhibited increased cone survival relative to vehicle-treated controls, with the greatest preservation observed in the combination treatment group (Figure S2). These findings suggest that simultaneously targeting distinct degenerative pathways provides enhanced protection against cone loss.

### 3.2. Inhibition of GALR3 and Quercetin Treatment Improve Retinal Function in rd10 Mice

**Figure 3:**
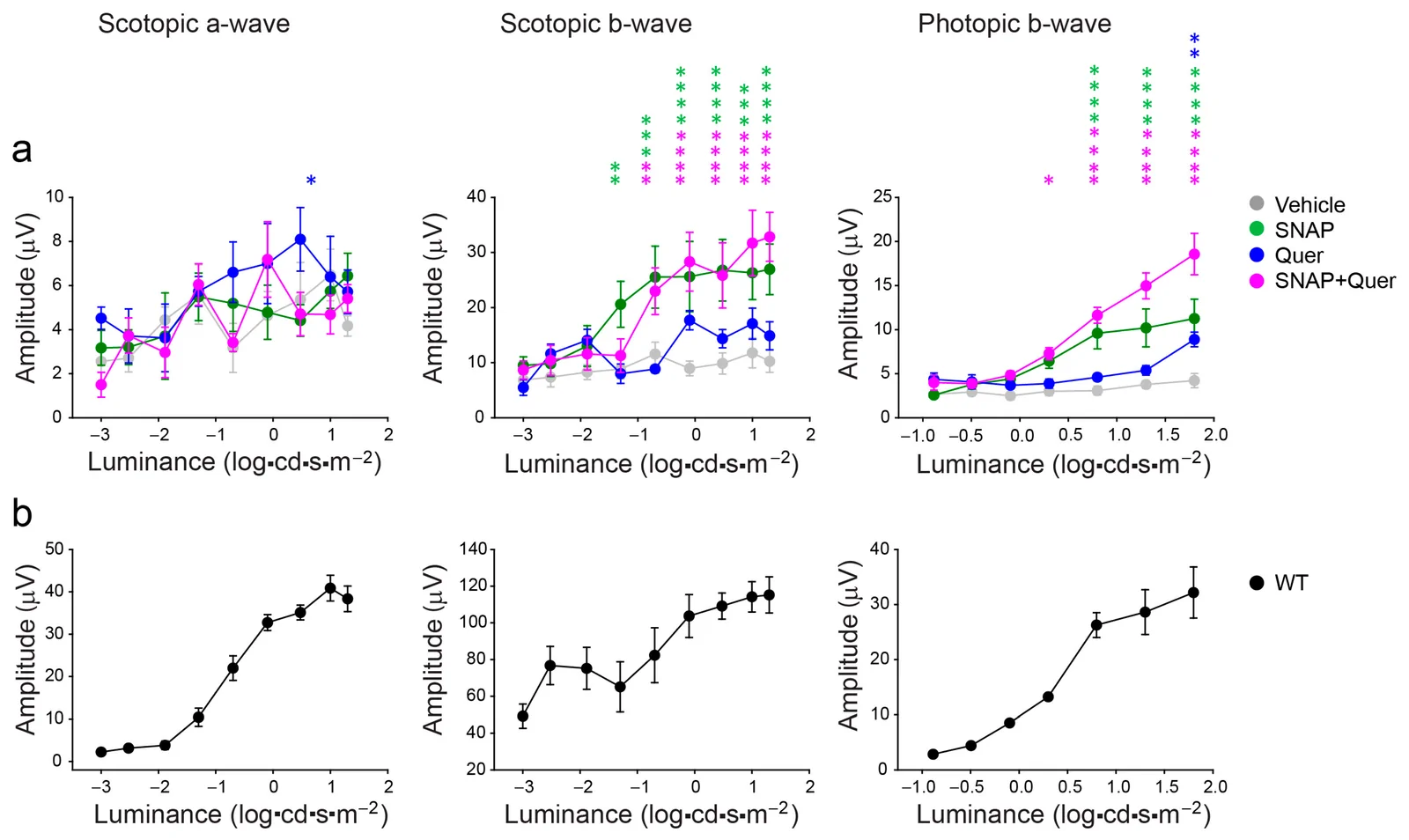
Effects of GALR3 antagonist, quercetin and their combination on retinal function in *rd10* mice. Retinal function was assessed with electroretinography (ERG) recordings. (**a**) ERG responses recorded in the following groups of mice: *rd10* vehicle-treated, *rd10* SNAP-37889-treated, *rd10* quercetin-treated, and *rd10*-treated with a combination of SNAP-37889 and quercetin. (**b**) ERG responses recorded in WT mice. Error bars represent standard error of the mean (SEM). Statistical analysis was performed with two-way ANOVA and post hoc Turkey’s tests. Statistically significant changes are shown with asterisks. *, *p* < 0.05; **, *p* < 0.01; ***, *p* < 0.001; ****, *p* < 0.0001.

To assess the effects of treatment on retinal function, we performed electroretinography (ERG). As expected, *rd10* mice exhibited markedly reduced retinal responses to increasing light intensities compared with WT controls. Given the primary defect in phototransduction, treatment elicited only modest improvements in scotopic rod a-wave responses. In contrast, greater improvements were observed in scotopic b-wave amplitudes and photopic cone responses, reflecting enhanced activity of secondary retinal neurons and improved cone function, respectively. Treatment with quercetin resulted in modestly increased scotopic b-wave and photopic b-wave amplitudes, whereas SNAP-37889 treatment produced more pronounced functional improvements. Notably, the combination of SNAP-37889 and quercetin resulted in the greatest enhancement of both scotopic b-wave and photopic responses, with photopic b-wave responses reaching nearly 50% of WT levels, indicating superior preservation of retinal function compared with either monotherapy (Figure 3a). Despite these improvements, ERG responses in all treatment groups remained below those observed in WT controls (Figure 3b).

### 3.3. Inhibition of GALR3 and Quercetin Treatment Attenuates Oxidative Stress Responses in rd10 Mice

Inhibition of GALR3 signaling and quercetin treatment have been shown to reduce oxidative stress and inflammatory responses triggered by chronic stress in P23H rhodopsin mutant mice, a common model of RP, resulting in partial preservation of photoreceptors [8,14,15]. In the present study, we investigated whether these interventions confer similar protective effects in *rd10* mice, a model of RP caused by a mutation in *Pde6b*, another gene critical for photoreceptor function. We further examined whether combined treatment with a GALR3 antagonist and quercetin provides greater neuroprotective benefits than either intervention alone.

**Figure 4:**
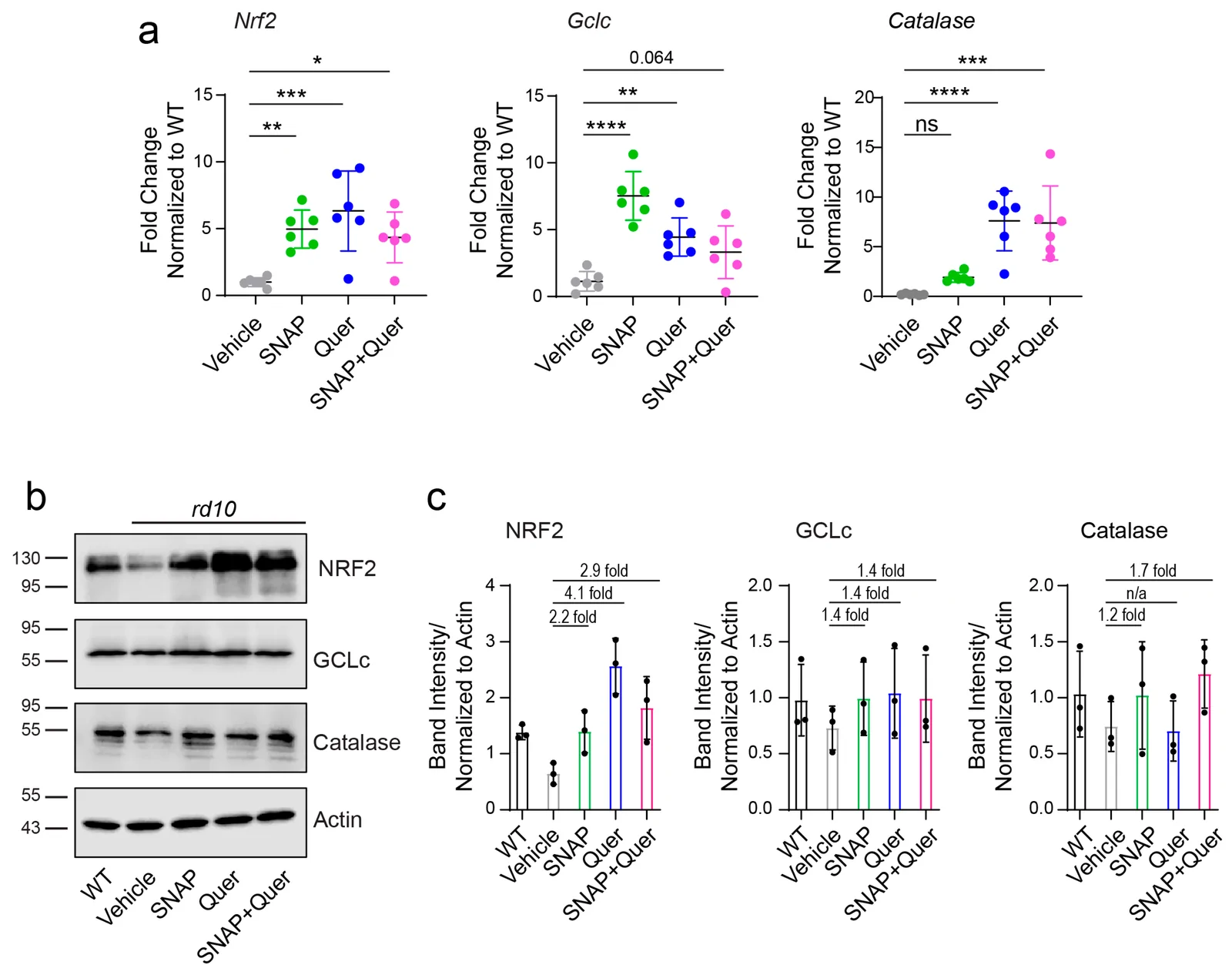
Effects of GALR3 antagonist, quercetin and their combination on oxidative stress in retinas of *rd10* mice. The analyses were performed in the following groups of mice: WT, *rd10* vehicle-treated, *rd10* SNAP-37889-treated, *rd10* quercetin-treated, and *rd10*-treated with a combination of SNAP-37889 and quercetin. (**a**) mRNA expression levels of antioxidant markers such as *Nrf2*, *Gclc*, and *catalase* detected with RT-qPCR normalized to *Gapdh* and these genes’ expression in WT mice. (**b**) Protein expression analysis of NRF2, GCLc, and catalase detected by immunoblotting. Representative immunoblots are shown. Full immunoblots are shown in Figure S2 in Supplementary Information. (**c**) Quantification of protein bands intensities’ detected from three independent immunoblot experiments, normalized to actin. Fold change was calculated. Error bars represent S.D. Statistical analysis was performed with one-way ANOVA and Dunnet’s post hoc tests. Statistically significant changes are shown with asterisks. *, *p* < 0.05; **, *p* < 0.01; ***, *p* < 0.001; ****, *p* < 0.0001; ns, not statistically significant.

Indeed, nuclear factor erythroid 2-related factor 2 (NRF2), a master regulator of the cellular antioxidant response [21,22], was significantly reduced in *rd10* retinas compared with WT controls. This decrease was accompanied by reduced mRNA and protein expression of its downstream target glutamate-cysteine ligase catalytic subunit (GCLc), the catalytic subunit of glutamate-cysteine ligase required for glutathione synthesis [23], as well as lower levels of the antioxidant enzyme catalase [24]. Following treatment, all three markers were significantly upregulated across treatment groups. NRF2 levels were restored, leading to a concomitant increase in GCLc expression and catalase levels, suggesting reactivation of the endogenous antioxidant defense system. These findings indicate that inhibition of GALR3 signaling and quercetin treatment enhance antioxidant capacity through NRF2 pathway activation, thereby promoting redox homeostasis and photoreceptor survival (Figure 4a–c and Figure S3) consistent with previously reported antioxidant and cytoprotective effects of quercetin [25,26]. Although single treatments were effective, no additive or synergistic effects were observed in the combination group, possibly due to convergence of both interventions on the same antioxidant pathway and saturation of the maximal protective response.

### 3.4. Inhibition of GALR3 and Quercetin Treatment Attenuates Inflammatory Responses in rd10 Mice

**Figure 5:**
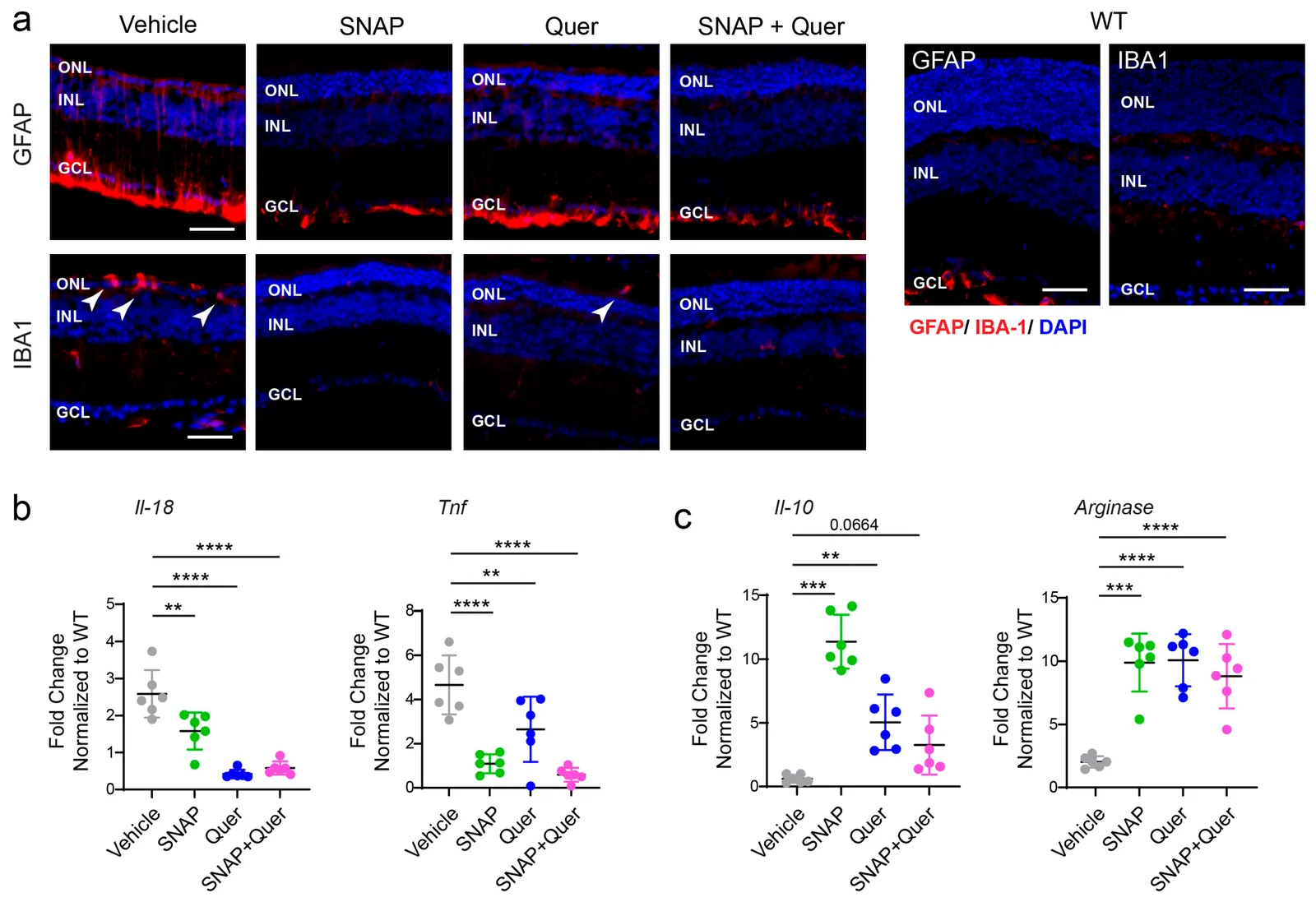
Effects of GALR3 antagonist, quercetin and their combination on the expression of pro-inflammatory and anti-inflammatory markers in retinas of *rd10* mice. The analyses were performed in the following groups of mice: WT, *rd10* vehicle-treated, *rd10* SNAP-37889-treated, *rd10* quercetin-treated, and *rd10*-treated with a combination of SNAP-37889 and quercetin. (**a**) Immunohistochemistry analysis on retinal cryosections detecting GFAP expression and localization (upper panel, red) and IBA1-positive cells (lower panel, red). Scale bar 50 µm. (**b**) mRNA expression levels of pro-inflammatory cytokines detected with RT-qPCR normalized to *Gapdh* and these genes’ expression in WT mice. (**c**) mRNA expression levels of anti-inflammatory markers detected with RT-qPCR normalized to *Gapdh* and these genes’ expression in WT mice. Error bars represent S.D. Statistical analysis was performed with one-way ANOVA and Dunnett’s post hoc tests. Statistically significant changes are shown with asterisks. **, *p* < 0.01; ***, *p* < 0.001; ****, *p* < 0.0001.

Two types of retinal resident glia, Müller glia and microglia, play critical roles in responding to retinal degeneration [27,28]. Müller glia, which extend radially across the retina, with their processes spanning from the inner limiting membrane to the outer limiting membrane, are among the first responders to photoreceptor injury [29]. Upon sensing photoreceptor degeneration, they initiate a reactive gliosis program characterized by upregulation of glial fibrillary acidic protein (GFAP) and secretion of signaling molecules that activate resident microglia. Activated microglia migrate to the outer retina to clear dying photoreceptors and maintain retinal health [30,31]. However, under chronic degenerative conditions driven by inherited mutations, this inflammatory response can become maladaptive and contribute to progressive photoreceptor loss [32]. Consistent with this, GFAP levels were elevated in *rd10* mice compared with WT controls, whereas treatment with the GALR3 antagonist, quercetin, or their combination attenuated reactive gliosis and reduced GFAP expression (Figure 5a).

In parallel, all treatment groups exhibited reduced proinflammatory signaling, as evidenced by decreased mRNA levels of interleukin-18 (*Il-18*) and tumor necrosis factor-α (*Tnf*) (Figure 5b). Conversely, the expression of anti-inflammatory markers, including *Il-10* and arginase, was increased following treatment, indicating a shift toward an anti-inflammatory retinal environment (Figure 5c). Together, these findings demonstrate that GALR3 inhibition and quercetin treatment attenuate both oxidative stress and inflammatory response, two major contributors to photoreceptor degeneration in *rd10* mice.

### 3.5. Inhibition of GALR3 Signaling and Quercetin Preserves Blood–Retina Barrier Integrity in rd10 Mice

The intact blood–retinal barrier (BRB) is essential for retinal homeostasis and photoreceptor metabolism [33]. The outer BRB, formed by retinal pigment epithelium (RPE) cells, and the inner BRB, formed by retinal capillary endothelial cells, rely on intact tight junctions to maintain homeostasis. In RP, BRB disruption occurs secondary to retinal remodeling driven by progressive photoreceptor degeneration, as previously reported in mouse models of RP [15,34]. In P23H rhodopsin mice, we previously observed that blocking GALR3 signaling mitigates this pathology [15], while the BRB-protective effects of quercetin have been shown in bright light-induced retinal damage models [34].

**Figure 6:**
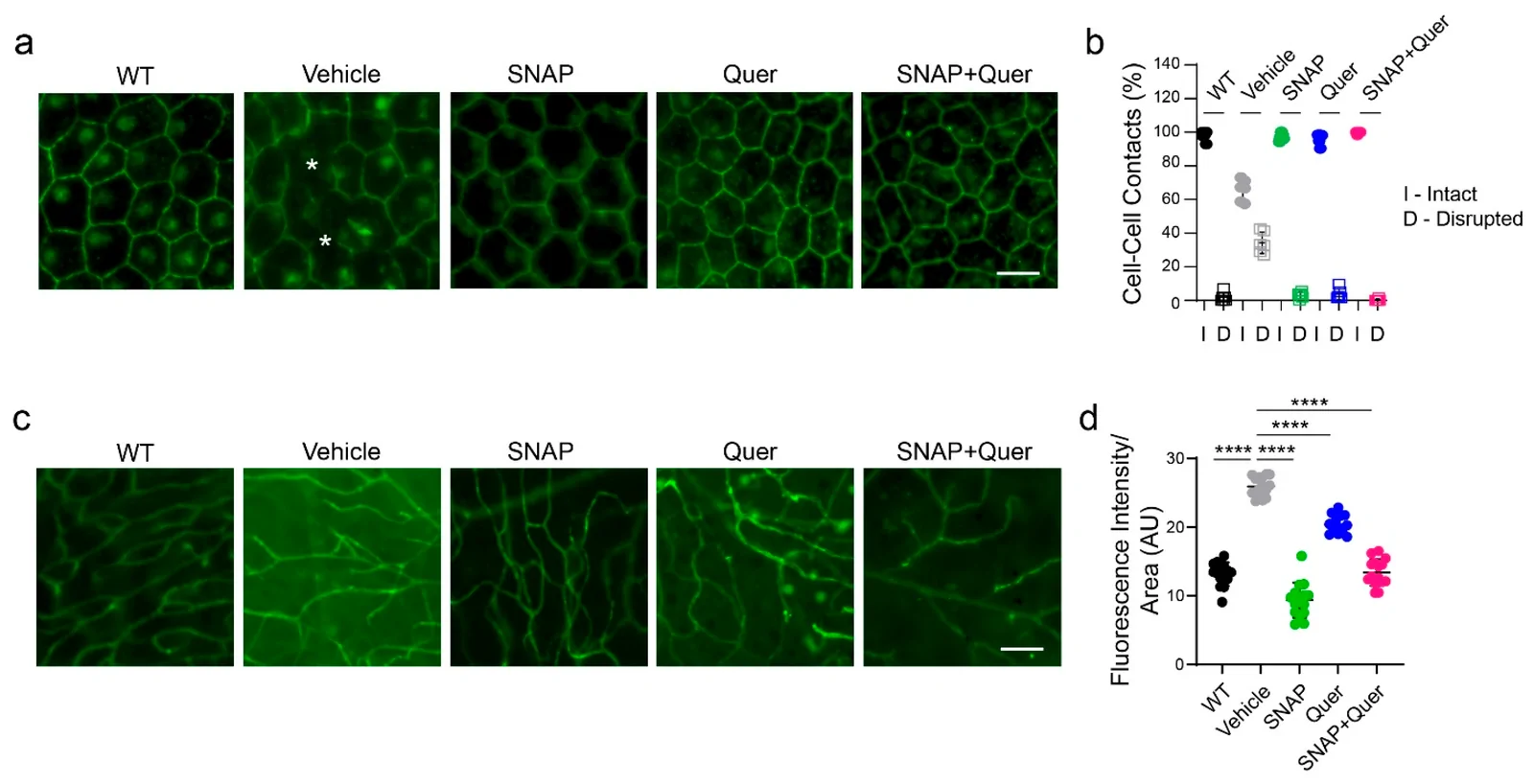
Effects of GALR3 antagonist, quercetin and their combination on BRB integrity in the eyes of *rd10* mice. The analyses were performed in the following groups of mice: WT, *rd10* vehicle-treated, *rd10* SNAP-37889-treated, *rd10* quercetin-treated, and *rd10*-treated with a combination of SNAP-37889 and quercetin. (**a**) Immunofluorescence labeling of ZO-1 on RPE-choroidal flat mounts. Pathological changes are shown with asterisks. (**b**) Quantification of RPE cell–cell connections per area shown in percent. (**c**) Detection of albumin leakage on retinal flat mounts with anti-albumin antibody conjugated to fluorescein. (**d**) Quantification of fluorescence intensity in retinal tissue. Error bars represent S.D. Statistical analysis was performed with one-way ANOVA and post hoc Dunnett’s tests. Statistically significant changes are shown with asterisks; ****, *p* < 0.0001.

In the present study, we found that tight junctions, visualized by ZO-1 staining, were disrupted in *rd10* mice. At P33, approximately 60% of retinal pigment epithelium (RPE) cells located in the retinal periphery exhibited loss of cell–cell junction integrity. In contrast, treatment with either the GALR3 antagonist or quercetin preserved RPE monolayer organization, preventing these pathological changes and maintaining a morphology comparable to WT controls (Figure 6a,b). To assess the functional consequences of this structural disruption, we evaluated vascular leakage using well-established FITC-conjugated anti-albumin immunostaining of retinal flat mounts [35,36,37,38] that we previously used to assess vascular leakage in P23H rhodopsin mice [15]. Under physiological conditions, albumin is restricted to the vasculature; however, disruption of the BRB results in its extravasation into retinal tissue. Consistent with BRB impairment, albumin leakage was detected in *rd10* mice, whereas no extravascular albumin signal was observed in any of the treatment groups (Figure 6c,d). Together, these findings demonstrate that GALR3 signaling contributes to BRB breakdown in *rd10* mice, consistent with observations in other models of retinal degeneration. Inhibition of GALR3 signaling, as well as quercetin-mediated reduction in oxidative stress, preserves both structural and functional integrity of the BRB, highlighting these treatments as potential therapeutic strategies for maintaining retinal homeostasis in degenerative retinal disease.

## 4. Discussion

Retinitis pigmentosa (RP) is an inherited retinal degenerative disorder in which progressive photoreceptor loss is driven not only by the initiating genetic defect but also by secondary pathological processes, including oxidative stress, neuroinflammation, mitochondrial dysfunction, and disruption of retinal homeostasis [4,5,39,40]. As photoreceptors degenerate, these secondary processes can become self-reinforcing, creating a pathological environment that further accelerates neuronal injury. An important therapeutic implication is that targeting a single downstream pathway may provide only partial protection, whereas simultaneous modulation of complementary pathological processes may provide broader neuroprotection. The major finding of the present study is that combined inhibition of GALR3 signaling and antioxidant treatment with quercetin provided greater protection of retinal structure and visual function than either treatment alone in the *rd10* mouse model of RP. These findings support a therapeutic strategy in which multiple secondary mechanisms contributing to retinal degeneration are targeted simultaneously rather than focusing exclusively on the causative genetic defect, consistent with recent evidence that combining gene-targeted and antioxidant therapies can enhance therapeutic efficacy and restore visual function in RP [41]. Similarly, another study in *rd10* mice has demonstrated that combination therapy targeting distinct pathogenic pathways, including GPCR-mediated cAMP/Ca^2+^ signaling and oxidative stress, can provide complementary therapeutic benefits, further supporting a multi-target approach to slowing photoreceptor degeneration [42].

The *rd10* mouse model recapitulates several important pathological features of human RP, including progressive photoreceptor loss, retinal remodeling, oxidative stress, neuroinflammation, and RPE dysfunction [43,44,45,46]. Consistent with previous reports, we found evidence of substantial oxidative and inflammatory stress in untreated *rd10* retinas, including reduced NRF2 signaling and decreased expression of downstream antioxidant enzymes, increased Müller glial reactivity and microglial activation, and elevated inflammatory cytokines. We also observed disruption of RPE organization and ZO-1-associated tight-junction integrity, accompanied by increased microglial accumulation in the outer retina. Together, these findings illustrate the complex pathological environment that develops during retinal degeneration and provide a rationale for targeting multiple secondary pathways.

Quercetin and GALR3 inhibition each provided significant protection against this pathological environment, although they are expected to influence different aspects of the disease process. Quercetin is a well-characterized flavonoid with antioxidant and anti-inflammatory properties that can reduce reactive oxygen species and enhance endogenous cellular defense mechanisms [8,26,47,48,49]. Consistent with these properties, quercetin treatment in our study was associated with preservation of retinal structure, reduced glial and inflammatory responses, and improved markers of retinal homeostasis. These findings are consistent with previous studies demonstrating protective effects of quercetin and related flavonoids in models of retinal degeneration, including *rd10* mice [8,50], and further support oxidative stress as an important modifiable component of photoreceptor degeneration.

In parallel, inhibition of GALR3 signaling produced a broadly similar protective phenotype, including preservation of retinal structure and reduction in inflammatory and glial responses. GALR3 is a G_i_-coupled GPCR expressed in the human retina [51] and has been implicated in stress-associated and neuroinflammatory signaling in the central nervous system [52,53] and other tissues [54,55]. Indeed, increased GALR3 expression has been reported in several pathological conditions, including Alzheimer’s disease [56], glioma [57], and stressed cardiac tissue [58], suggesting a broader role in disease-associated signaling. We previously demonstrated increased GALR3 expression and signaling in the P23H rhodopsin model [14,15]. The finding that GALR3 is similarly upregulated in *rd10* retinas, despite the distinct initiating defect in PDE6B, further supports its involvement in a common stress- and inflammation-associated response across genetically distinct forms of RP. Thus, GALR3 inhibition may represent a mutation-independent therapeutic strategy that targets downstream pathways contributing to retinal degeneration rather than the primary genetic defect itself.

The most important finding of the present study, however, was the enhanced protection produced by combining GALR3 inhibition with quercetin. Whereas each monotherapy produced beneficial effects, combined treatment resulted in greater preservation of retinal structure and improved ERG responses, with particularly notable effects on photopic visual function consistent with enhanced preservation of cone photoreceptors. These findings indicate that the benefit of combining the two interventions extends beyond molecular changes in individual stress-response pathways and is reflected in functionally meaningful preservation of the retina. Interestingly, the enhanced structural and functional protection produced by combined treatment was not accompanied by a consistently additive effect on the individual oxidative stress and inflammatory markers examined in this study. This apparent dissociation is important when interpreting the potential mechanism of the combination treatment. Oxidative stress and neuroinflammation are highly interconnected and dynamic processes [4,59], and GALR3 signaling and quercetin may influence overlapping downstream pathways [8,15]. Consequently, substantial attenuation of these pathways by either intervention may limit the extent to which further changes in individual endpoint biomarkers can be detected following combined treatment. In addition, the molecular markers assessed in the present study represent selected components of complex cellular responses, whereas cone survival and ERG measurements integrate the cumulative effects of multiple pathological and protective processes over the course of retinal degeneration. Thus, the greater preservation of cone photoreceptors and improvement in ERG responses following combined treatment should not be interpreted as evidence that the combination necessarily produces additive suppression of each measured oxidative or inflammatory marker. Rather, the enhanced functional benefit may reflect complementary effects of the two interventions on multiple aspects of retinal homeostasis that are not fully captured by the selected biomarkers. For example, GALR3 inhibition and quercetin may influence distinct upstream or downstream processes affecting cellular resilience, photoreceptor metabolism, mitochondrial function, or neuronal survival. Although the present study does not establish the precise molecular mechanism responsible for the enhanced functional response, the dissociation between individual biomarkers and functional outcomes emphasizes that molecular and functional measures provide complementary information about therapeutic efficacy.

The translational significance of these findings lies in the potential to develop treatment strategies that are less dependent on the underlying RP mutation. More than 70 genes have been implicated in RP [3,60,61], making mutation-specific therapeutic development challenging, particularly for patients carrying rare or less common disease-causing variants. In contrast, secondary processes such as oxidative stress and neuroinflammation are shared features of many forms of retinal degeneration and may therefore provide broader therapeutic entry points [62,63]. Our findings suggest that combining pharmacological modulation of disease-associated GALR3 signaling with an antioxidant intervention can simultaneously target multiple convergent mechanisms that contribute to photoreceptor loss. Such an approach could potentially complement, rather than replace, emerging gene- and mutation-specific therapies by reducing the secondary cellular damage that persists after initiation of the primary genetic insult.

The use of the genetically distinct mouse model is particularly relevant to this translational concept. Our previous studies established a protective role for GALR3 inhibition in P23H rhodopsin-associated degeneration [14,15], whereas the present study demonstrates that GALR3 inhibition is also protective in *rd10* degeneration caused by a defect in PDE6B. The observation of a similar protective response in these mechanistically distinct models strengthens the possibility that GALR3-dependent signaling represents a convergent component of retinal degeneration rather than a pathway specific to *RHO*-associated disease. Furthermore, the enhanced efficacy achieved by combining GALR3 inhibition with quercetin demonstrates the potential value of targeting multiple secondary mechanisms simultaneously. These findings extend our previous work from demonstrating the involvement of GALR3 in retinal degeneration to providing a pharmacological rationale for a combination strategy directed at complementary pathogenic pathways.

An additional consideration is the clinical relevance of preserving cone function. Although rod photoreceptors are typically affected earlier in RP, progressive cone degeneration is a major contributor to severe visual impairment, particularly the loss of central and high-acuity vision [64]. The greater preservation of photopic ERG responses and cone photoreceptors observed with combined treatment therefore suggests that targeting secondary stress pathways may have functional consequences beyond simply delaying overall photoreceptor loss. Whether such protection can be maintained over longer periods and translate into preservation of visual behavior remains to be determined. Long-term studies will be particularly important because the therapeutic value of a mutation-independent strategy will ultimately depend on its ability to preserve meaningful visual function during the chronic course of disease.

Several limitations should also be considered. Although combined treatment produced greater structural and functional protection than either monotherapy, the present study was not designed to establish the precise molecular interaction between GALR3 signaling and quercetin-responsive antioxidant pathways. In particular, the absence of additive responses in individual oxidative and inflammatory markers does not allow us to conclude that the two pathways are mechanistically independent or that their combined effect is synergistic. Additional studies will be required to determine whether enhanced protection reflects complementary or additive actions and to identify the downstream mechanisms responsible. Dose–response studies, formal pharmacological interaction analyses, longer treatment periods, pharmacokinetic studies, and evaluation at later stages of degeneration will also be important for assessing the translational potential of this approach.

## 5. Conclusions

Collectively, our study demonstrates that GALR3 inhibition and quercetin each mitigate pathological changes associated with retinal degeneration, while their combination provides greater preservation of retinal structure, cone photoreceptors, and visual function than either intervention alone. Importantly, the enhanced functional benefit was not accompanied by uniformly additive changes in the oxidative and inflammatory markers examined, suggesting that therapeutic efficacy cannot be inferred solely from individual molecular endpoints. Instead, the findings support a model in which simultaneous modulation of interconnected secondary stress pathways can promote retinal resilience through complementary mechanisms. The results showing the GALR3-dependent protection in both P23H rhodopsin and *rd10* mouse models further support GALR3 as a potential mutation-independent therapeutic target. More broadly, these findings provide a proof-of-concept that combination strategies directed at convergent secondary mechanisms may complement mutation-specific therapies and offer a broader therapeutic approach for slowing photoreceptor degeneration and preserving visual function in RP.

## Figures and Tables

**Table 1:** List of used commercial antibodies.

Antibody Name	Species	Source	Identifiers	Additional Information
Anti-M-Opsin	Rabbit polyclonal	Millipore	AB5405 RRID:AB_177456	1:1000
Anti-GFAP	Rabbit polyclonal	Thermo Fisher Scientific	PA1-9565 RRID:AB_2109797	1:1000
Anti-IBA1	Rabbit polyclonal	Thermo Fisher Scientific	PA5-27436 RRID:AB_2544912	1:100
Anti-Catalase	Rabbit polyclonal	Abclonal	A11220 RRID:AB_2861525 8018	1:1000
Anti-GCLc	Rabbit polyclonal	Invitrogen	PA5-103134 RRID:AB_2852504	1:500
Anti-NRF2	Mouse monoclonal	Santa Cruz	Sc-365949 RRID:AB_10917561	1:1000
Anti-Albumin FITC conjugated	Rabbit polyclonal	Thermo Fisher Scientific	A90-234F RRID:AB_67126	1:400
Anti-Actin	Mouse monoclonal	Thermo Fisher Scientific	MA1-744 RRID:AB_2223496	1:1000
Anti-mouse IgG, HRP conjugate	Goat	Promega	W4021 RRID:AB_430834	1:10,000
Anti-rabbit IgG, HRP conjugated	Goat	Promega	W4011 RRID:AB_430833	1:10,000
Anti-mouse IgG Alexa Fluor 555-conjugated	Goat	Thermo Fisher Scientific	A28180 RRID:AB_2536164	1:400
Anti-rabbit IgG Alexa Fluor 555-conjugated	Goat	Thermo Fisher Scientific	A27039 RRID:AB_2536100	1:400

**Table 2:** List of used primers.

Target	Accession Number	Species	Forward Primer Sequence 3′→5′	Reverse Primer Sequence 3′→5′
*Rhodopsin*	NM_145383.2	mouse	CTTCCTGATCTGCTGGCTTC	ACAGTCTCTGGCCAGGCTTA
*M-opsin*	NM_008106.2	mouse	GAGATTCAAGAAGCTGCGCC	TGTCCAGAACGAGTAGCC
*Crx*	NM_007748.4	mouse	TACCTACAATCCCATGGACC	CTTTGGCAGGATTGCTACTT
*Il-10*	NM_010548.2	mouse	AGGCGCTGTCATCGATTTCT	TGTTACACTCGCCCCCTTTG
*Il-18*	NM_008360.2	mouse	TTACAAGCATCCAGGCACAG	GAAGGTTTGAGGCGGCTTTC
*Tnf*	NM_013693.3	mouse	GGTCTGGGCCATAGAACTGA	CAGCCTCTTCTCATTCCTGC
*Gclc*	NM_008128.4	mouse	GCTTTGGGTCGCAAGTAGGA	GCGTCCCGTCCGTTCC
*Catalase*	NM_009804.2	mouse	ACCACACATCCTGAACGAGGAG	GATGAAGCAGTGGAAGGAGC
*Nrf2*	NM_010902.5	mouse	ATCTCCTAGTTCTCCGCTGC	CAAAACTTGTACCGCCTCGT
*Gapdh*	NM_008084.3	mouse	TTGAGGTCAATGAAGGGGTC	TCGTCCCGTAGACAAAATGG
*Galr3*	NM_008086.3	mouse	TCGTGTGCAAGACGGTACA	ACCGCCAGGTACCTATCCA
*Spexin*	NM_001285487.1	mouse	CGCCTCCAGAAAGACGAAAC	AATTCCCTCCTTCATCTGCACC

## Data Availability

The data that support the findings of this study are included in the manuscript and Supplementary Information.

## Supplementary Materials

The following supporting information can be downloaded at https://www.mdpi.com/article/10.3390/antiox15091073/s1, Figure S1: Full immunoblots for Figure 2b; Figure S2: Cone photoreceptor density analysis; Figure S3: Full immunoblots for Figure 4b.

## Abbreviations

The following abbreviations are used in this manuscript:

arRP	Autosomal recessive retinitis pigmentosa
BRB	Blood–retina barrier
BSA	Bovine serum albumin
ERG	Electroretinography
GALR3	Galanin receptor 3
GPCR	G protein-coupled receptor
GFAP	Glial fibrillary acidic protein
GCLc	Glutamate-cysteine ligase catalytic subunit
IL-10	Interleukin 10
IL-18	Interleukin 18
NGS	Normal goat serum
NRF2	Erythroid 2-related factor 2
Pde6b	Phosphodiesterase 6b
PFA	Paraformaldehyde
PNA	Peanut agglutinin
ROS	Reactive oxygen species
RPE	Retinyl pigment epithelium
SD-OCT	Spectral domain-optical coherence tomography
TNF-α	Tumor necrosis factor alpha
WT	Wild type
