# Supplementary material for: Dual Targeting of Galanin Receptor 3 Signaling and Redox Homeostasis Enhances Photoreceptor Survival in Retinas of rd10 Mice
Source: Antioxidants (Basel). 2026 Aug 27;15(9):1073. doi: 10.3390/antiox15091073 (PMC13603368; doi:10.3390/antiox15091073)
Supplement: Supplementary file 1 [file antioxidants-15-01073-s001.zip › antioxidants-4473271-supplementary.pdf]

Supplementary Information

Dual targeting of GALR3 signaling and redox homeostasis enhances photoreceptor survival in retinas of *rd10* mice

Maria Azam[1], Mingda Liu[1], and Beata Jastrzebska[1,2*]

[1]Department of Pharmacology, School of Medicine, Case Western Reserve University, 10900 Euclid Ave., Cleveland, OH 44106, USA

[2]Cleveland Center for Membrane and Structural Biology, School of Medicine, Case Western Reserve University, 10900 Euclid Ave., Cleveland, OH 44106, USA

[*] **CORRESPONDENCE**

Beata Jastrzebska, Ph.D., Department of Pharmacology, School of Medicine, Case Western Reserve University, 10900 Euclid Ave., Cleveland, OH 44106-4965, USA; Phone: 216-368-5683; Fax: 216-368-1300; E-mail: bxj27@case.edu; ORCID ID: https://orcid.org/0000-0001-5209-8685.

Keywords: galanin receptors, neuroinflammation, oxidative stress, photoreceptor, quercetin, retinal degeneration, retinitis pigmentosa

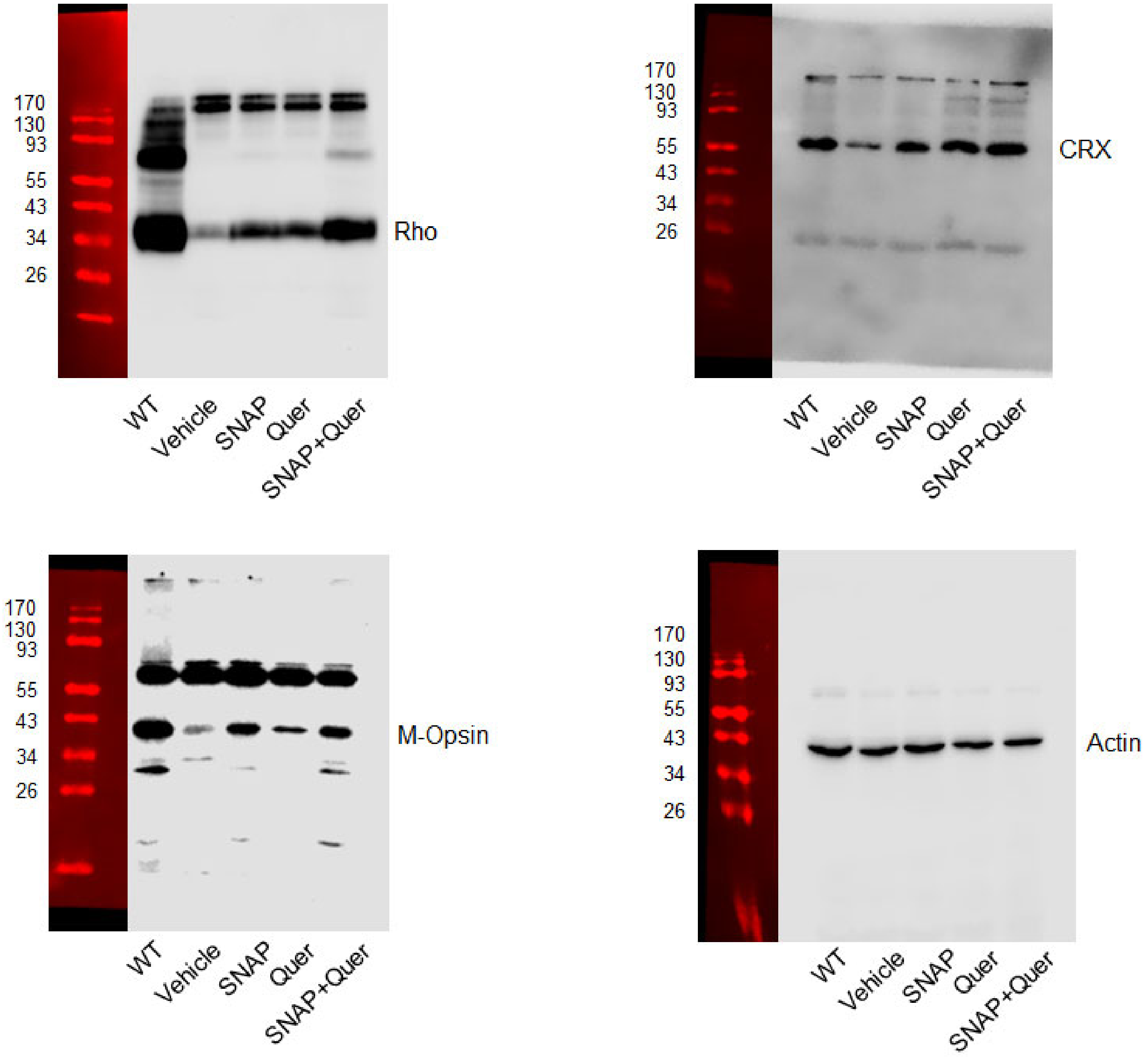

Figure S1. Full immunoblots for Fig. 2b.

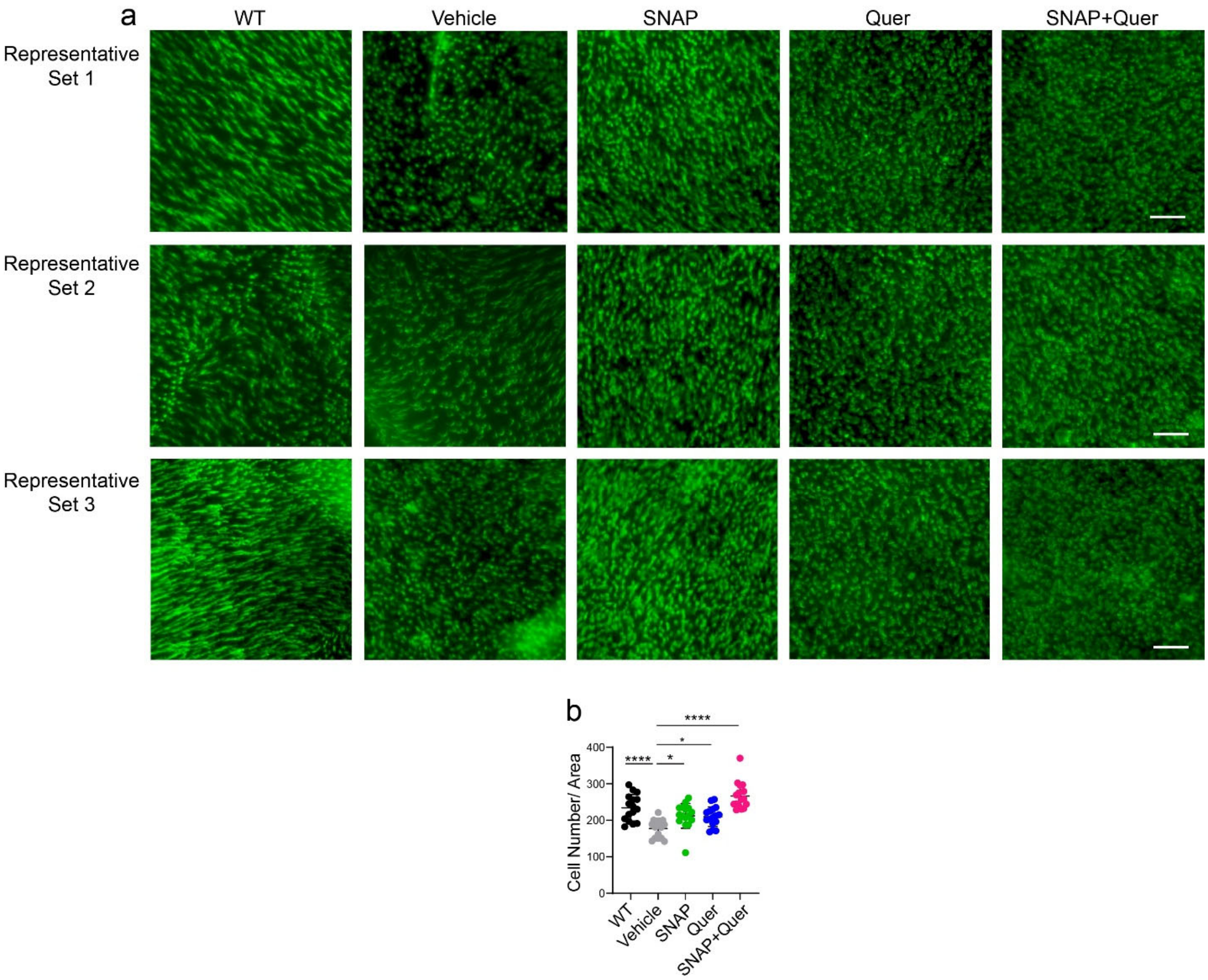

Figure S2. Cone photoreceptors density analysis. a, Images of retinal flat mounts labeled with PNA to detect cone photoreceptors. Three representative images per treatment group are shown. Images were taken at the retinal periphery at 20x magnification. **b**, Quantification of cone photoreceptor number. Cone count per area is shown. Scale bar 20 µm. Error bars represent S.D. Statistical analysis was performed with one-way ANOVA and post hoc Dunnet's tests. Statistically significant changes are shown with asterisks. *, $P<0.05$; **, $P<0.01$; ***, $P<0.001$; ****, $P<0.0001$; ns, not statistically significant changes.

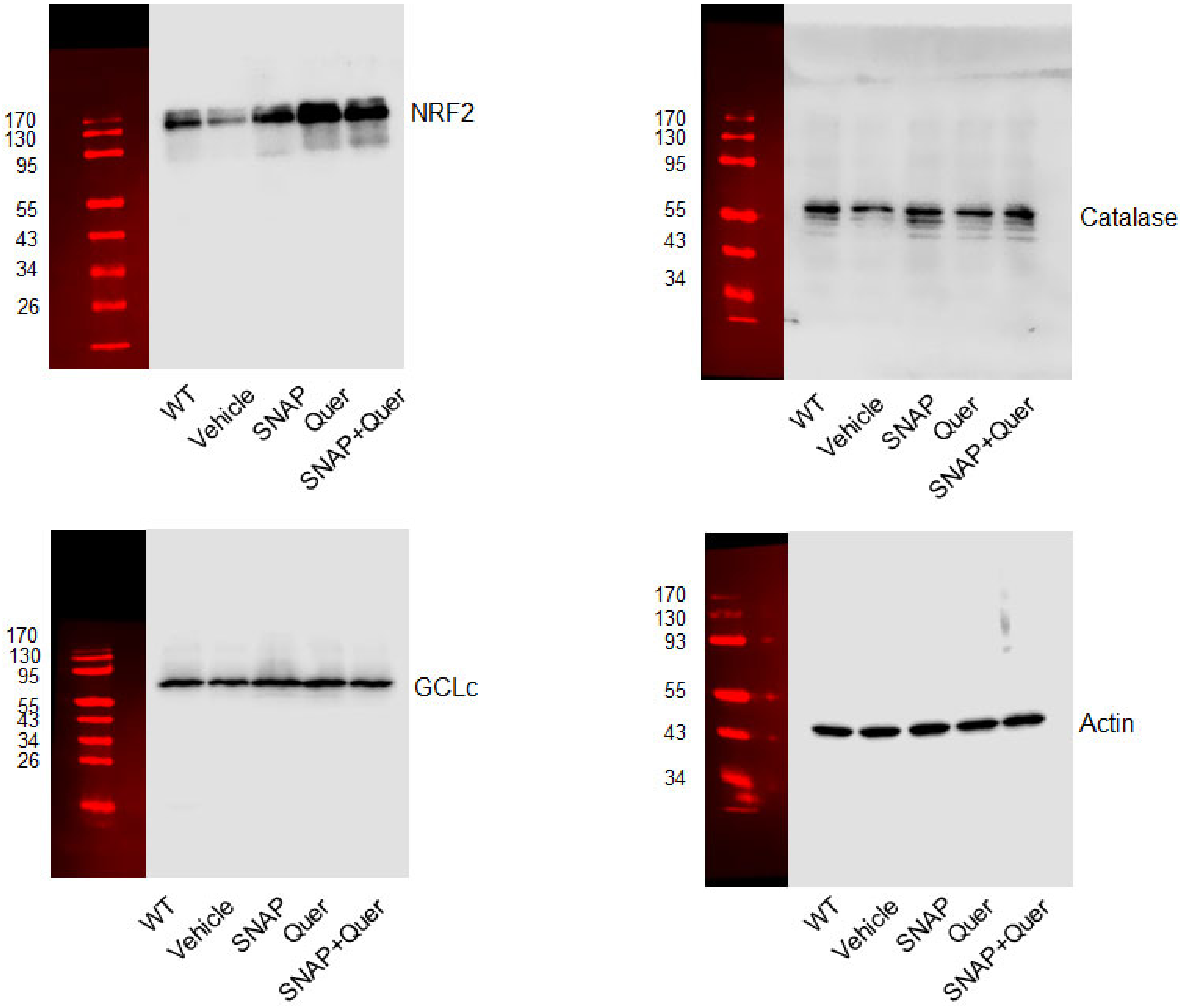

Figure S3. Full immunoblots for Fig. 4b.
